# Two-stage dual-game model approach to view the difficulty of healthcare accessibility

**DOI:** 10.3389/fpubh.2023.1078675

**Published:** 2023-03-09

**Authors:** Weiwei Wang, Futian Weng, Yusheng Chen, Miao Zhu

**Affiliations:** ^1^School of Medicine, Xiamen University, Xiamen, China; ^2^National Institute for Data Science in Health and Medicine, Xiamen University, Xiamen, China; ^3^Data Mining Research Center of Xiamen University, Xiamen University, Xiamen, China; ^4^Finance and Economics College, Jimei University, Xiamen, China; ^5^School of Statistics, Huaqiao University, Xiamen, China

**Keywords:** El Farol Bar problem, game theory, learning theory, Nash equilibrium, healthcare accessibility

## Abstract

This study proposed a two-stage dual-game model methodology to evaluate the existing difficulty of healthcare accessibility in China. First, we analyzed a multi-player El Farol bar game with incomplete information by mixed strategy to explore the Nash equilibrium, and then a weighted El Farol bar game was discussed to identify the existence of a contradiction between supply and demand sides in a tertiary hospital. Second, the overall payoff based on healthcare quality was calculated. In terms of the probability of medical experience reaching that expected level, residents are not optimistic about going to the hospital, and the longer the observation period is, the more pronounced this trend becomes. By adjusting the threshold value to observe the change in the probability of being able to obtain the expected medical experience, it is found that the median number of hospital visits is a key parameter. Going to the hospital did bring benefits to people with consideration of the payoffs, while the benefits varied significantly with the observation period among different months. This study is recommended as a new method and approach to quantitatively assess the tense relationship in access to medical care between the demand and supply sides and a foundation for policy and practice improvements to ensure the efficient delivery of healthcare.

## 1. Introduction

Healthcare resources are always limited compared to the demand of residents. With increasing healthcare burdens, such as aging populations and growing prevalence of communicable or non-communicable diseases (1), how to efficiently allocate limited healthcare resources is gaining increasing attention globally, and the idealized way of allocating resources is to match available supply with demand (2). However, this is a multi-sector game involving patients, medical institutions, doctors, and relevant departments (3), which can be summarized as the demand side, supply side, and regulating side of healthcare services. In China, the healthcare service system was different from other countries, which has formed a system of universal medical insurance and covers more than 95% of Chinese citizens, including three major insurance programs, namely, Urban Employee Basic Medical Insurance, Urban Resident Basic Medical Insurance, and New Rural Cooperative Medical Insurance. From the perspective of delivery, China's healthcare delivery system is fragmented and hospital-centered, constituted of four major categories of providers, namely, hospitals, primary healthcare institutions, professional public health organizations, and others (e.g., physician clinics) (4). Hospitals are designated as primary, secondary, or tertiary institutions based on a three-tiered grade system, and further subdivided into three subsidiary levels according to the size, staff and equipment, and medical quality. Due to a large population together with disease burden transitions over the past few decades (5), China experienced an increasing demand for healthcare, which placed significant pressure on its healthcare system to become more accessible, affordable, and efficient (6).

From the supply side, China has made significant investments in its healthcare services, and the total health expenditure increased from CNY 1,998.39 billion in 2010 to CNY 6,519.59 billion in 2019, with a proportional increase from 11.03 to 17.93% (7). Moreover, the healthcare workforce increased from 8,207,502 to 12,928,335 over the vsame period, while the number of healthcare institutions increased from 936,927 in 2010 to 1,007,579 in 2019 (8). From the demand side, the demand for healthcare services increased due to disease burden transitions and an aging population; moreover, the majority of the population tends to seek primary care at large tertiary hospitals, which has resulted in the highly concentrated allocation of supply-side resources at tertiary public hospitals. Gaps in health-related investment and investment-output efficiency are of the first importance in the research of fundamental mismatch between supply and demand sides as well as other drivers related to this issue. On the one hand, the number of tertiary hospitals was 2,996 in 2020, accounting for 8.46% of hospitals. On the other hand, tertiary hospitals engage in 54.21% of outpatient services and 51.07% of inpatient services (9). Health service delivery is organized as highly centralized in the Chinese healthcare system and relies on rigid institutional arrangements (10). Since there are few, if any, gatekeepers to services in hospitals, it is common for tertiary hospitals to provide basic outpatient services in addition to broader research and advanced medical services. This expansive service provision, combined with greater public trust in larger public hospitals over local health clinics, has overburdened public hospitals, resulting in significant systemic inefficiencies (11, 12).

Healthcare resources are always limited compared to demand, and how to most efficiently allocate limited healthcare resources is gaining increasing attention. The ultimate goal of the healthcare supply chain is to meet the demands of healthcare requirements, and it is usually practically synonymous between hospitals and the concept of a healthcare delivery system (13). A growing body of literature has studied the use of healthcare services and the factors associated with utilization. Spurred by the desire to help ensure that all members of society would make optimal use of healthcare, medical and public health researchers and behavioral and social scientists have been increasingly drawn to the study of human behavior and health (14). In non-emergency situations, the last experience attending medical care has an influence on the next medical decision to a certain extent. Given the fact that the mismatch between the supply and demand sides of medical resources is particularly prominent in tertiary hospitals, whether those in healthcare will change their decision-making to avoid resource contention is a research area of practical significance.

The purpose of this article is to show, using a two-stage dual-game model, how an individual chooses the oncoming medical-seeking behavior given previous attendance experience and medical service capacity. In previous studies, researchers usually set the payoffs of going to a hospital as a fixed value and discuss the relationship between the probability of patients going to the hospital and the payoffs, without consideration of influences from others. To deal with this gap effectively, we propose a new, dynamic dual-game model. Specifically, we construct an El Farol Game model to evaluate the payoff of healthcare seeking a tertiary hospital, thus forming a dual-game effect that allows the original model to estimate the payoff for going to the hospital, with full consideration of interactions between these two non-independently game models. Moreover, the reinforcement learning was also adopted as a supplement to the methodology, which has been broadly applied in optimizing decision-making issues (15, 16) and has a special potential in sequential decision-making in the context of healthcare domains (17). The El Farol Bar problem can be seen as a prototypical model of distributed resource allocation in which a given agent's utility depends on the number of other agents who choose to utilize the same resource (18). Most of the literature on the El Farol Bar problem and its derivatives is devoted to the search for decision rules in the use of the resource. Taking a tertiary hospital in China as an example, we focus on the antagonism between demand and supply sides, capturing the influence of medical resources contradiction on people's tendency to seek healthcare in tertiary hospitals combined with the consideration of healthcare quality measured through a composite healthcare payoff index.

## 2. Materials and methods

### 2.1. Data sources

The data used for analysis in this study were collected from two sources. The number of daily outpatient visits to a tertiary hospital for January–December 2020, as well as information on the healthcare quality index, including bed occupancy rate, average stay length of discharged patients, total number of treatments, number of admissions, average daily hospital physician visits, and average daily number of inpatient bed days for hospitalized patients, were collected from Hospital-X, one of the major tertiary hospitals in Zhangzhou, Fujian Province, which integrates medical treatment, teaching, and research. The population migration in this city is relatively low and the choice over healthcare facility for residents is relatively stable, which facilitates the following analysis by excluding spatial factors and is more suitable for analytic structure consequently. The other data source is the Health and Healthcare Development Statistics Bulletin, from which we obtained the national healthcare service data as a complement.

### 2.2. Analysis

#### 2.2.1. El Farol Bar game

We consider a generalized El Farol Bar game which was proposed by Arthur (19) to demonstrate his point that perfect rationality and a logical apparatus have limits in dealing with complicated problems. In this generalized El Farol Bar game, there are *N* players. Each player can choose one of the *k* actions in the action set {0, 1, …, *k*−1}. Those players who choose action zero always receive a payoff of zero. Player *i* who chooses action *j* receives a payoff of *w*_*ij*_*H*, if the sum of all actions played by the *N* players is less than or equal to c. Otherwise, player *i* who chooses action *j* receives a payoff of −*w*_*ij*_*L*. We assume that *H*>0 and *L*>0. The payoffs are summarized in Table 1.

**Table 1:** Payoffs of a generalized El Farol Bar game (H > 0 and L > 0).

**Actions**	**Payoffs if the sum of actions is less than or equal to c**	**Payoffs if the sum of actions is greater than c**
0	0	0
1	w_ij_H	-w_ij_L

At first, we analyze a multi-player El Farol bar game according to Tirole (20), in which there are *N* players and each player assumes that the other player adopts a mixed strategy. Each player maintains a belief probability distribution for the other players with equal weights in order to calculate his/her expected payoff for each action. Specifically, let *x*_*i*_(*t*) be the fraction of time that player *i* takes action 1 in time interval (0, t), and this information is known to everyone in this game. Each player, except the player *i*, believes that player *i* adopts a mixed strategy, in which player *i* takes action 1 with probability *x*_*i*_(*t*) and action 0 with probability 1−*x*_*i*_(*t*).

Now, we consider the fictitious play of a specific player *i*. Let *u*_*ij*_(*t*) denote *i*'s expected payoff by taking action *j* and {*X*_1_, *X*_2_, ⋯ , *X*_*N*_} be a sequence of independent Bernoulli random variables. Specifically,


$$P(X_{k}=j)=\{\begin{array}{cc} x_{k} & j=1\\ 1-x_{k} & j=0 \end{array}$$

The event {*X*_*k*_ = *j*} denotes player *i*'s belief that player *k* will take action *j* in *k*'s mixed strategy. Based on this belief, we can analyze *i*'s expected payoff. First, we define *u*_*i*, 0_ = 0. In addition,


$$\begin{array}{l} u_{i,1}=HP\left(\sum_{k=1,k\neq i}^{N}X_{k}+1\leq c\right)-LP\left(\sum_{k=1,k\neq i}^{N}X_{k}+1>c\right)\\ \;=\text{H}-\left(\text{H}+\text{L}\right)\text{P}\left(\sum_{k=1,k\neq i}^{N}X_{k}\geq c\right) \end{array}$$

*u*_*i*, 0_ ≤ *u*_*i*, 1_ if and only if


(1) $$\begin{array}{l} P\left(\overset{N}{\underset{k=1,k\neq i}{\sum }}X_{k}\geq c\right)\leq u \end{array}$$

where


$$\begin{array}{l} u=\frac{H}{H+L} \end{array}$$

Clearly, *u* is strictly between zero and one. Now, rewrite the condition in Eq. (1). Let *p* = {1, 2, ⋯ , *N*} be the set of all players and *p*_−*i*_ = {1, 2, …, *i*−1, *i*+1, …, *N*} with an exclusion of play *i*. Now, consider subset *S*′ of set *p*_−*i*_. The number of elements in *S*′ equals to *j*. That is, |*S*′| = *j*. We have


(2) $$\begin{array}{l} P\left(\sum_{k=1,k\neq 1}^{N}X_{k}\;\geq \;c\right)\;=\;\sum_{j=c}^{N}P\left(\sum_{k=1,k\neq 1}^{N}X_{k}\;=j\right)\\ \;=\;\sum_{j=c}^{N-1}\sum_{\forall S^{\prime }\subseteq p_{-i}}\prod_{k\in S^{\prime }}x_{k}(t)\prod_{l\in p_{-i}-S^{\prime }}\left(1-x_{k}(t)\right) \end{array}$$

where the second summation on the right side of Eq. (2) can be evaluated by enumerating all possibilities


(3) $$\begin{array}{l} \overset{N-1}{\underset{j=c}{\sum }}\underset{\forall S^{\prime }\subseteq p_{-i}}{\sum }\underset{k\in S^{\prime }}{\prod }x_{k}\left(t\right)\underset{l\in p_{-i}-S^{\prime }}{\prod }\left(1-x_{k}\left(t\right)\right)<u \end{array}$$

Player *i* will take action 1 in the fictitious play if inequality (3) holds. We can derive a balance equation for the number of times that action 1 is taken in time interval (0, t). Specifically,


$$\begin{array}{l} \left(t+1\right)x_{i}\left(t+1\right)=tx_{i}\left(t\right)+I\left\{u_{i,0}\left(t\right)\leq u_{i,1}\left(t\right)\right\} \end{array}$$

where *I*{*E*} is an indicator function of event *E*. If event *E* occurs, *I*{*E*} = 1. Otherwise, *I*{*E*} = 0. We can approximate this balance by a differential equation


$$\begin{array}{l} {x^{\prime }}_{i}\;(t)\;=\;\frac{x_{i}(t)}{t+1}\;+\;\frac{1}{t+1}I\left\{u_{i,0}(t)\;\leq \;u_{i,1}\;(t)\right\}\\ \;=\frac{x_{i}(t)}{t+1}\;+\;\frac{1}{t+1}I\left\{\sum_{j=c}^{N-1}\sum_{\forall S^{\prime }\subseteq p_{-i}}\prod_{k\in S^{\prime }}x_{k}(t)\prod_{l\in p_{-i}-S^{\prime }}(1-x_{k}(t))\;\leq \;u\right\} \end{array}$$

*i* = 1, 2…, *N* is an on-autonomous dynamical system.

Recall that the value of an indicator function is either 1 or 0. Thus, the entries of x^*^ are either 1 or 0. Let *k* be the number of unity entries in vector x^*^. We can conclude that *k* cannot be 0, nor can be N. Suppose *k* = 0, then the *i*-th entry of x^*^, denoted by ${x_{i}}^{*}$, is $x_{i}^{*}=0$. In this case, the product of entries which correspond to any subset of *p*_−*i*_ is zero. Thus, inequality Eq. (3) holds, since μ is strictly between 0 and 1, which implies that $x_{i}^{*}=1$, and brings contradictions with the assumption that $x_{i}^{*}=0$. In contrast, if *k* = *N*, $x_{i}^{*}=1$ for some players, since ${x_{i}}^{*}$ is an N-1 dimensional vector, it is easy to check that all products on the left side of Eq. (3) are 0, except the product corresponding to the case in which *j* = *N*− 1 and $S^{\prime }=p_{-i}$ with the product 1. It follows that $x_{i}^{*}=0$, which contradicts with the assumption. Thus, we conclude that 1 ≤ *k* ≤ *N*− 1.

Since *k* < 1, there is at least one unity entry in x^*^. Assume that $x_{i}^{*}=1$ for some players, it follows that for each player, Eq. (3) must hold and the corresponding indicator function gives the value 1. Thus,


(4) $$\begin{array}{l} \underset{\forall S\subseteq p_{-i}}{\sum }\underset{k\in S^{\prime }}{\prod }x_{k}^{\text{*}}\left(t\right)\underset{l\in p_{-i}-S^{\prime }}{\prod }\left(1-x_{k}^{\text{*}}\left(t\right)\right)=0 \end{array}$$

or any *j* = {*c, c*+1, …, *N*−1}. For Eq. (4) to hold, there must be at least (*N*−1)−*c*+1 zero entries in the vector $x_{i}^{*}$. Hence,


$$\begin{array}{l} k\leq 1+\left(N-1\right)-\left(N-1-c+1\right)=c \end{array}$$

In contrast, since k <N-1, there is at least one zero entry in x^*^, thus,


(5) $$\begin{array}{l} \underset{\forall S\subseteq p_{-i}}{\sum }\underset{k\in S^{\prime }}{\prod }x_{k}^{\text{*}}\left(t\right)\underset{l\in p_{-i}-S^{\prime }}{\prod }\left(1-x_{k}^{\text{*}}\left(t\right)\right)=1 \end{array}$$

there are at least c unity entries in vector ${x_{i}}^{*}$. Suppose that there are n <c unity entries in ${x_{i}}^{*}$, the only nonzero term in the summation on the left side corresponds to the product in which *j* = *n* and set *S*′ contains exactly the indexes of all the unity entries in ${x_{i}}^{*}$. All other values of *j* and selections of *S*′ contribute zero to the sum on the left side of Eq. (5). Since $x_{i}^{*}=0$, it follows that


$$\begin{array}{l} k=n\geq c \end{array}$$

We note that x^*^ corresponds to a Nash equilibrium of pure strategies.

Let *x*_*ij*_ = *A*_*ij*_(*t*)/*t* for player *i* and action *j*, while *X*_*i*_ be a random variable whose probability mass function is


$$\begin{array}{l} P\left(X_{i}=j\right)=A_{ij}\left(t\right)/t \end{array}$$

for *i* = 1, 2, …*N*, and *j* = 0, 1, …*k*−1. To analyze the fictitious play process of this generalized El Farol Bar game, we assume that each player aggregates the belief probability distribution of individual opponents and maintains an aggregated belief probability distribution. For example, player *i* maintains the probability mass function of


$$\begin{array}{l} Y_{i}=X_{1}+X_{2}+\cdots +X_{i-1}+X_{i+1}+\cdots +X_{N} \end{array}$$

Player *i* determines the best action by calculating expected payoffs using the probability mass function of *Y*_*i*_. Specifically, *u*_*i*, 0_(*t*) = 0. Furthermore,


(6) $$\begin{array}{l} u_{ij}(t)\;=w_{ij}HP(Y_{i}+j<c)-w_{ij}LP(Y_{i}+j>c)\\ \;=w_{ij}(H+L)(\frac{H}{H+L}-P(Y_{i}\geq c-j+1)) \end{array}$$

Denote the probability mass function of *Y*_*i*_ by


$$\begin{array}{l} y_{ij}=P\left(Y_{i}=j\right) \end{array}$$

For *i* = 1, 2, …*N* and *j* = 0, 1, …(*N*−1)(*k*−1), we rewrite Eq. (6) as


(7) $$\begin{array}{l} u_{ij}\left(t\right)=w_{ij}\left(H+L\right)\left(\frac{H}{H+L}-\sum_{k=c-j+1}^{\left(N-1\right)\left(k-1\right)}y_{ik}\right) \end{array}$$

One can derive a set of balance equations for the number of times that actions are taken by each player. That is,


(8) $$\begin{array}{l} \left(t+1\right)x_{ij}\left(t+1\right)=tx_{ij}\left(t\right)+I\left\{j=argmax\left\{u_{ik}\left(t\right)\right\}\right\} \end{array}$$

One can approximate the above set of difference equations by the following system of differential equations:


(9) $$\begin{array}{l} x_{ij}^{{}^{\prime }}\left(t+1\right)=\frac{-x_{ij}\left(t\right)}{t+1}+\frac{1}{t+1}I\left\{j=argmax\left\{u_{ik}\left(t\right)\right\}\right\} \end{array}$$

for *i* = 1, 2, …, *N*, and *j* = 0, 1, …, *k*−1.

Now, we consider the equilibrium point of the system in Eq. 9. For fixed *i* and *j*, let $x_{ij}^{*}$ be the equilibrium point and denote vector $x_{ij}^{*}$ as $\left\{x_{i,0}^{*},x_{i,1}^{*},\ldots ,x_{i,k-1}^{*}\right\}$. Since {*x*_*ij*_(*t*):0 ≤ *j* ≤ *k*−1} is a probability distribution, exactly one indicator function in the set of equations for each specific *i* and 0 ≤ *j* ≤ *k*−1 is one. Other indicator functions are zero. That is, for each *i*, there is an action *j*(*i*) correspondingly. The value of the indicator function corresponding to *j*(*i*) is one and others are all zero. We can construct it as follows:


(10) $$x_{ij}^{*}=\{\begin{array}{cc} 1 & j=j(i)\\ 0 & \;otherwise\; \end{array}$$

Define


(11) $$y_{ij}^{*}=\{\begin{array}{cc} 1 & j=j^{*}\\ 0 & \;otherwise\; \end{array}$$

where $j^{*}=\sum_{k=1,k\neq j}j\left(k\right)$.

Substituting (6) into (1), we can obtain


$$u_{ij}(t)=\{\begin{array}{cc} -w_{ij}L & c-j+1\leq j\\ w_{ii}H & \;otherwise\; \end{array}$$

It can be seen that *u*_*ij*_(*t*) achieves the maximum value when *j* = *c*−j^*^. Thus, we have


(12) $$\begin{array}{l} j\left(1\right)+j\left(2\right)+\cdots +j\left(N\right)=c \end{array}$$

Eq. (12) corresponds to a Nash equilibrium of fixed strategies.

Here, we can see that the bar problem is very similar to the scenario of a patient going to a hospital for treatment. Each hospital has a certain capacity limit. When the number of patients exceeds a certain level, each patient will receive medical benefits less than the normal, which brings the mindset that it is difficult to access healthcare for the public. To clarify this problem, we apply a dual-game model consisting of hospital-pharmacy and hospital–patient to reflect the existence of healthcare access problem for the local population.

#### 2.2.2. Reinforcement learning

Reinforcement learning is a branch within machine learning that is adept at controlling an individual that can act autonomously in a given environment and continuously improve its behavior by interacting with the environment. Reinforcement learning problems include learning what to do and how to map the environment into actions to maximize reward (21). In this article, we introduce reinforcement learning to solve the game problem.

**Figure 1:**
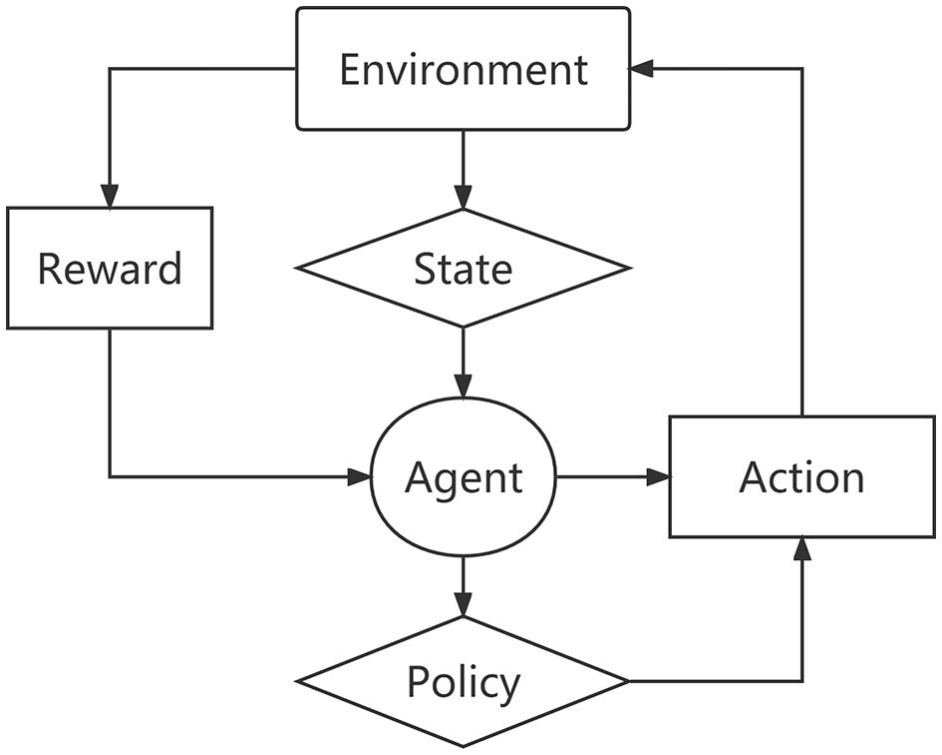
The framework of reinforcement learning.

Reinforcement learning consists of an agent, an environment, a state, an action, a reward, and a policy (22, 23). As shown in Figure 1, the strategy determines the agent's behavior at a given time, thereby mapping the current state of the environment into actions that correspond to a set of the so-called stimulus-response rules in psychology.

Specifically, the reinforcement learning elements can be expressed as:


D={S,A,ℚ,D,γ}

where *S* is the state set space; *A* is the set space for actions; ℝ denotes the reward obtained by the agent after taking action according to the state; ℙ is the transition probability matrix of state action; γ denotes the return discount rate, and the value is between 0 and 1.

Suppose the sequence generated by the interaction between the agent and the environment is:


$$\begin{array}{l} \tau =\left\{s_{0},a_{0},r_{0},s_{1},a_{1},r_{1},\ldots \right\} \end{array}$$

The return of the agent at time *t* is *r*_*t*_, and then the total return after time *t* is


$$\begin{array}{l} G_{t=0:T}=R_{\tau }=r_{t+1}+r_{t+2}+\cdots +r_{T} \end{array}$$

Definitely, the strategy is the mapping of the action probability distribution π(*a*|*s*) of each state *s*∈*S* and action *a*∈*A*, that is, the probability of the agent taking action *a* in state *s* is


$$\begin{array}{l} \pi \left(a|s\right)=p\left(A_{t}=a|S_{t}=s\right),\exists t \end{array}$$

Therefore, given the initial state distribution ρ_0_ and the strategy π, the probability of occurrence of a *T*-step trajectory τ in the Markov process is


$$\begin{array}{l} p\left(\tau |\pi \right)=\rho_{0}\left(S_{0}\right)\prod_{t=0}^{T-1}p\left(S_{t+1}|S_{t},A_{t}\right)\pi \left(A_{t}|S_{t}\right) \end{array}$$

Finally, expected return *J*(π) can be defined as


$$J\left(\pi \right)=\int_{\tau }p(\tau |\pi )R\left(\tau \right)={\mathbb{R}}_{\tau \sim \pi }\left[R\left(\tau \right)\right]$$

Here, *R* denotes the reward function; *p*(τ|π) indicates the probability of occurrence of the trajectory. Reinforcement learning can maximize returns by optimizing strategies and then solve the above game problems.

#### 2.2.3. Dual-game model approach to healthcare-seeking behavior analysis

As the basic component of this dual-game model, we have a need to depict the hospital-pharmacy game at first. We set the probability of going to a hospital as *p*_0_ and the payoff *E* in the situation, while the probability of going to a pharmacy as 1−*p*_0_ and the payoff *e*. Then, we set the payoff as 0 for the case of neither going to a hospital nor a pharmacy and the payoff as *E*+*e* for the case of both going to the pharmacy and hospital. The expected payoff E is determined by the El Farol game model, where the initial probability in the model is *p*_0_. Thus, the expected payoff equation in the El Farol game model will become an important parameter of the hospital-pharmacy model. At the same time, these two game models are not independent but interrelated. As demonstrated earlier, the hospital-pharmacy model is based on the underlying game theory model, and we will focus our research on hospital–patient game.

In reality, data on the “difficulty of healthcare accessibility” has a time-dimension feature. Thus, for each day, each individual who has a latent need for medical treatment is involved in a “hospital–patient” game. Notably, we need to make some reasonable assumptions about our model. First, we define the optimal number of hospital visits as *c*. Second, we define the probability *p*, which represents the probability that a patient believes the current number of hospital visits is less than the optimal number *c*. Third, the probability *p* is updated for each patient based on previous hospital visits for a day or some days. For the update method here, we apply Markov decision processes (MDPs), which can be simply expressed as *M* = {*S, A, R, w*}, where

1. The set *S* is the set of probabilities *p*.
2. The set *A* is the set of finite actions; here, we set it as the patient's self-predicted behavior after viewing previous experience. The behavior here is mainly for the patient to self-renew “the probability of receiving good treatment at the hospital.”
3. The set *R* represents the immediate reward of a patient taking an action (i.e., reward). *R* is set to 0 and 1; 1 represents the number of hospital visits less than the expected number of patients, and 0 is the opposite.
4. *w* is denoted as the learning rate.

We introduce the concept of discount rate into this function so that the reward returned from future states is multiplied by this discount coefficient. This implies that the present reward is more important than the reward returned in the future, which is also more intuitive. Finally, we can write the whole system of MDPs as the following two equations:

A balance equation is


(13) $$\begin{array}{l} p_{t}=\left(1-\omega \right)p_{t-1}+\omega \times R\times I\left\{Action_{t}\right\} \end{array}$$

where *p*_*t*−1_ denotes the probability of choosing to go to the hospital at time *t*−1; *Action*_*t*_ and *p*_*t*_ express the action and the probability of choosing to go to the hospital at time *t* separately.

For the balance equation, we need to obtain an expression for its convergence to a steady state. Since the event *S* is probabilistic events, it satisfies the basic requirements of probability. We assume that the initial probability *p*_1_ = *p*, then


$$\begin{array}{l} p_{2}=(1-\omega )p+\omega \times R\times I\{Action_{2}\}\\ p_{3}=(1-\omega )p_{2}+\omega \times R\times I\{Action_{3}\}\\ \;\;\;\;\;\;={\left(1-\omega \right)}^{2}p+(1-\omega )\times \omega \times R\times I\{Action_{2}\}\\ \;\;\;\;\;\;\;\;\;\;+\omega \times R\times I\{Action_{3}\}\\ \vdots \\ p_{t}={\left(1-\omega \right)}^{t-1}p+{\left(1-\omega \right)}^{t-2}\times \omega \times R\times I\{Action_{2}\}+\cdots \\ \;\;\;\;\;+\omega \times R\times I\{Action_{t}\} \end{array}$$

Therefore, we can determine the range of *p*_*t*_ as follows:


$$\begin{array}{l} p_{t}={\left(1-\omega \right)}^{t-1}p+{\left(1-\omega \right)}^{t-2}\times \omega \times R\times I\{Action_{2}\}+\cdots \\ \;\;\;\;\;\;+\omega \times R\times I\{Action_{t}\}\\ p_{t}\geq {\left(1-\omega \right)}^{t-1}p\\ p_{t}\leq {\left(1-\omega \right)}^{t-1}p+{\left(1-\omega \right)}^{t-2}\omega +\cdots +\omega \\ \;\;\;\;\;\;={\left(1-\omega \right)}^{N-1}p+\omega \frac{\left(1-{\left(1-\omega \right)}^{t-1}\right)}{1-(1-\omega )}\\ \;\;\;\;\;\;=1-{\left(1-\omega \right)}^{t-1}(1-p)\\ \;\;\;\;\;\;\leq 1 \end{array}$$

The other equation is the expected payoff equation:


(14) $$\begin{array}{l} E=Hp-L\left(1-p\right) \end{array}$$

Recall that a “hospital–patient” game with N players. Each player can choose one of the two actions: 0 or 1. A patient who chooses the action “not see a doctor” always receives a payoff of 0. If a patient chooses the action “see a doctor”, then the payoff he or she receives depends on the sum of the actions of all patients. If the sum is less than or equal to *c*, the player receives a payoff of *H*. Otherwise, the player receives a payoff of *L*. We assume that *H*>0 and *L*>0. The notation used throughout the main text is summarized in Supplementary Table S1.

## 3. Results

### 3.1. Emergency situation

The outpatients are usually separated into emergency and general outpatients according to the emergency degree. Emergency care, which is intended for patients who are in critical illness, deserves prioritization in life-saving treatments (24) and in medical resource allocation, it is significantly different from the general situations, which requires us to analyze these two different situations separately. Here, we carry on the analysis of emergency outpatients at first. Due to the specificity of emergency patients, we chose *p* = 1 and one observation day for model analysis, and we also chose the average daily number of emergency outpatients in Hospital-X as the threshold value. The experimental results are shown in Table 2.

**Table 2:** Change in *p*-value in emergency situations for 12 months.

**Month**	**Update *p*-value (average)**	**Month**	**Update *p*-value (average)**
Jan	0.968	Jul	0.813
Feb	1	Aug	0.806
Mar	0.994	Sep	0.9
Apr	0.993	Oct	0.845
May	0.9613	Nov	0.827
Jun	0.82	Dec	0.845

We can find that the variation of *p* for emergency situations ranges between 0.8 and 1, with an average value of 0.897, demonstrating the insufficiency of healthcare resources impeded healthcare accessibility for emergency outpatients for many months, while the willingness for individuals seeking urgent healthcare services to a tertiary hospital remained close to 1. Here again, as the emergency model is a special case of the outpatient model, and the volume of outpatient care is much higher than that of emergency for the main body of hospital operations, we will focus on the outpatient model in the following sections.

### 3.2. Balance equation

#### 3.2.1. Overall analysis

We used the daily number of visits to Hospital-X in 2020 for analysis. First, we set the threshold *c*=1,250 based on the average number of daily visits in 2020 (mean: 1,238.88). Second, we chose the learning efficiency as 0.2. Third, we set the initial value of probability *p* as 0.5 according to our selection of different observation strategies for the updated *p*-value. Finally, we obtained the *p*-value change in 2020 (refer to Table 3).

**Table 3:** The overall situation with probability under changed observation days.

**Day(s) of observation**	**The mean probability**	**Number of days with *p* greater than 0.5 (*n* = 366)**	**Percentage of days with *p* greater than 0.5 (*n* = 366)**
1 day	0.481	149	0.408
2 days	0.467	149	0.408
3 days	0.455	139	0.381
4 days	0.446	116	0.318
5 days	0.439	116	0.318
6 days	0.433	97	0.266
7 days	0.428	94	0.258

**Figure 2:**
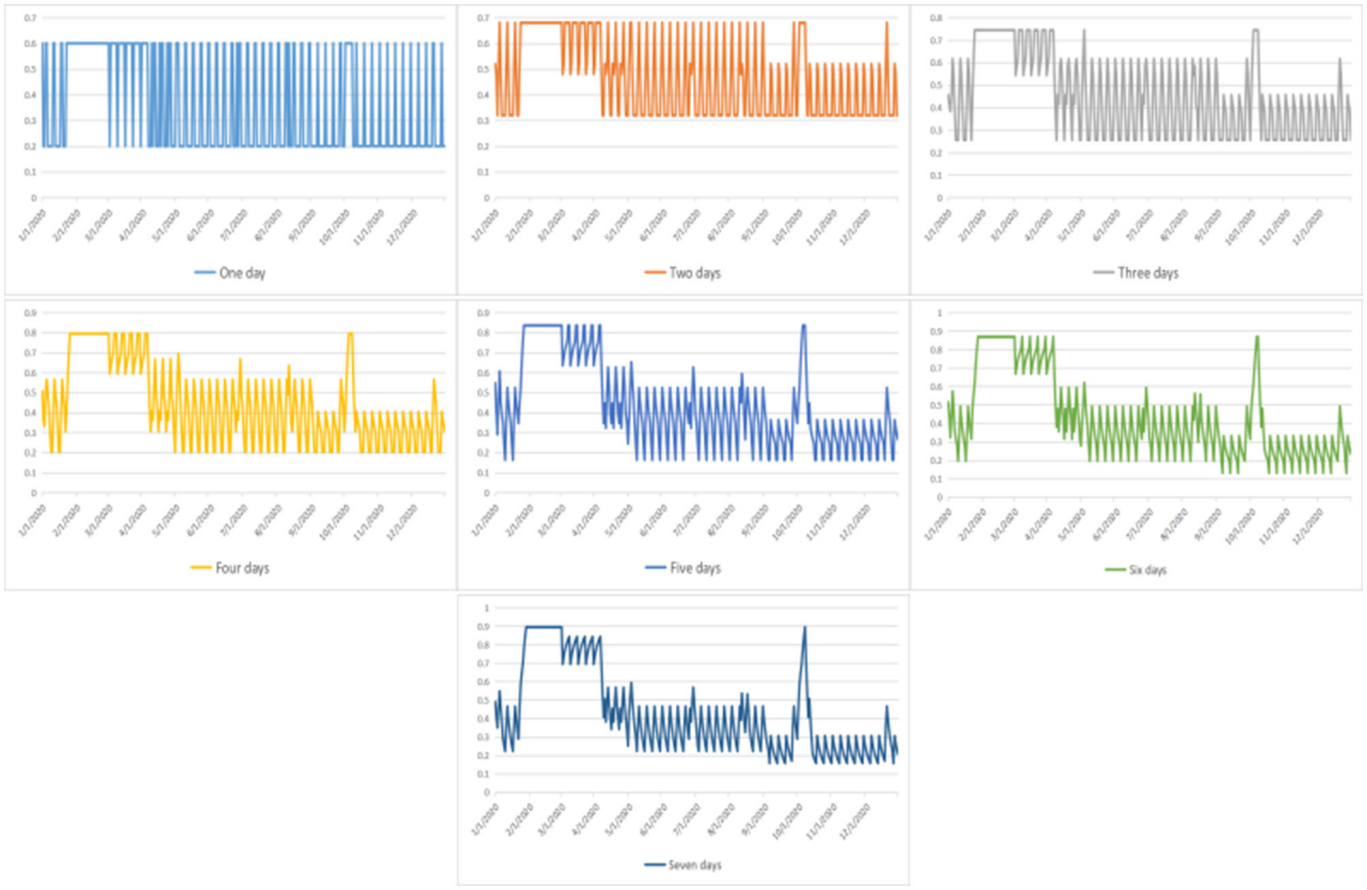
The probability that a patient believes the current number of hospital visits is less than the threshold under changed strategies.

**Figure 3:**
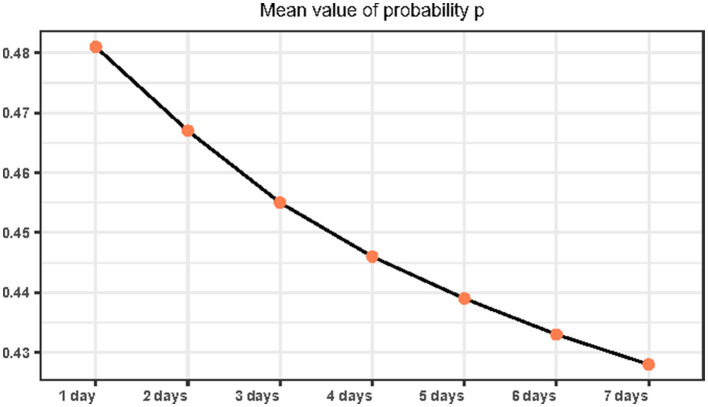
Mean of the probability that a patient believes the current number of hospital visits is less than the threshold under changed strategies.

In Figure 2, we can see that when the observation strategy is >3 days, the change in the probability *p* of patients perceiving the number of hospital admissions to be less than the limit *c* shows the same trend. Therefore, in our subsequent simulations and studies, we will select the three strategies for the observation of 1, 2, and 3 days as the main object of analysis. Here, we choose to analyze the overall situation with the specific values presented in Table 3. We can also see that in 2020, with the increase in the number of days in the observation strategy, the average value and the trend of *p* obviously show a decreasing trend (refer to Figure 3).

Simultaneously, the number of days with a probability *p* greater than 0.5 also shows an obviously decreasing trend. Moreover, we find that regardless of the number of observation days, the number of days with a probability *p* > 0.5 does not exceed 180 days. As such, we found that under the current conditions, the public does not show optimism about good access to healthcare, which reflects the public's perception of difficulty in healthcare access. In addition, we found that people are not optimistic about good access to healthcare under the current conditions, which reflects the “difficulty of healthcare accessibility”. Simultaneously, we found that when people receive more information about medical care, it will aggravate their perception of the difficulty in healthcare accessibility.

Consider the fact that physician-specific aspects are usually outstanding in the field of most valuable considerations for patients (25). As the physician scheduling is usually fixed by the day of the week for outpatient services in China, especially for specialists, here outpatient numbers on different days of the week are extremely heterogeneous with respect to physician scheduling. As shown in Supplementary Table S2, compared to weekends, the weekdays are more likely to be overwhelmed with heavy outpatient loads and bring down the belief probability of receiving a preferred health service. Moreover, we can find heterogeneity even within the workdays, and the lowest belief probability comes on Monday as most specialists are scheduled and there is a surge in demand due to the weekend accumulation.

#### 3.2.2. Setting of initial p-value

According to the above analysis, we can find that the *p*value decreases with observation days and needs detailed discussion. Here, we set a range of values from 0.3 to 0.7 as initial *p*, and observe the changes in our model at different *p*-value sets. First, we set *p* = [0.3, 0.7] and three observation days with a learning coefficient of 0.2 to observe the variation pattern of the final *p*-value. Second, we set *p* = [0.3, 0.7] and seven observation days with a learning coefficient of 0.2 to observe the variation pattern of the final *p*-value. The specific data are shown in Tables 4, 5.

**Table 4:** The variation pattern of final *p*-value for 3 days.

**Original**	**Update *p*-value (average)**	**Rate of change**
0.3	0.353	18%
0.4	0.404	1%
0.5	0.455	−9%
0.6	0.506	−16%
0.7	0.557	−20%

**Table 5:** The variation pattern of final *p*-value for 7 days.

**Original**	**Update *p*-value (average)**	**Rate of change**
0.3	0.386	29%
0.4	0.407	2%
0.5	0.428	−14%
0.6	0.449	−25%
0.7	0.470	−33%

We can see an interesting phenomenon from the above two sets of data. The value after updating will become higher than the initial value by our updating algorithm when *p* < 0.5, while the contrary happens when *p*≥ 0.5. Moreover, the adjustment amplitude is smallest at the set of *p* = 0.4, and the further away from 0.4, the greater the adjustment amplitude is. Furthermore, we approached the Nash equilibrium point infinitely by Newton's method to be 0.41. Meanwhile, the more observation days and the more information obtained in our set, the greater the *p*-value changes after the update.

#### 3.2.3. Discount rate

Our goal is to measure the difficulty of healthcare accessibility and provide evidence for improving the status. As such, we need to consider the impact of past experiences on the probability *p* at first and we changed the discount rate to observe the trend of the mean probability *p*. According to the previous data analysis, three observation strategies of 1, 3, and 5 days and a threshold *c* of 1,250 were selected for further analysis.

**Figure 4:**
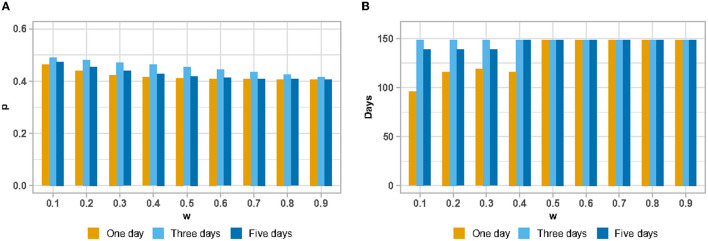
The mean value of probability that a patient believes the current number of hospital visits is less than the threshold under change of discount rate (w: discount rate; p: probability) **(A)**; The number of days with the probability that a patient believes the current number of hospital visits is less than the threshold >0.5 **(B)**.

According to Eq. (6), we can find that the influence of action will become more important as the discount rate increases, which makes the objective factors play a dominant role. First, we start with the threshold c = 1,250 when the population observes that hospital visits are more stressful than relaxing. In this condition, the mean value of *p* shows a decreasing trend with an increase in the discount rate. The strategy of observing 1 day was most significantly affected by the discount. Simultaneously, we find that when the discount rate γ increases, the discount rate of the strategy of observation days becomes less pronounced and the final *p*-means will tend to be the same, as shown in Figure 4A.

Statistics for the number of days with a probability *p*> 0.5 show some interesting scenarios (refer to Figure 4B). With γ = 0.5 as the dividing line, the number of days with probability *p* > 0.5 shows an inverse effect as the number of days in the observation strategy increases when γ <0.5. Under the situation of λ>0.5 and objective factors appearing in a dominant position, we find that the observation strategy will not affect the statistic of the number of days with a probability *p* > 0.5.

#### 3.2.4. Threshold value

**Figure 5:**
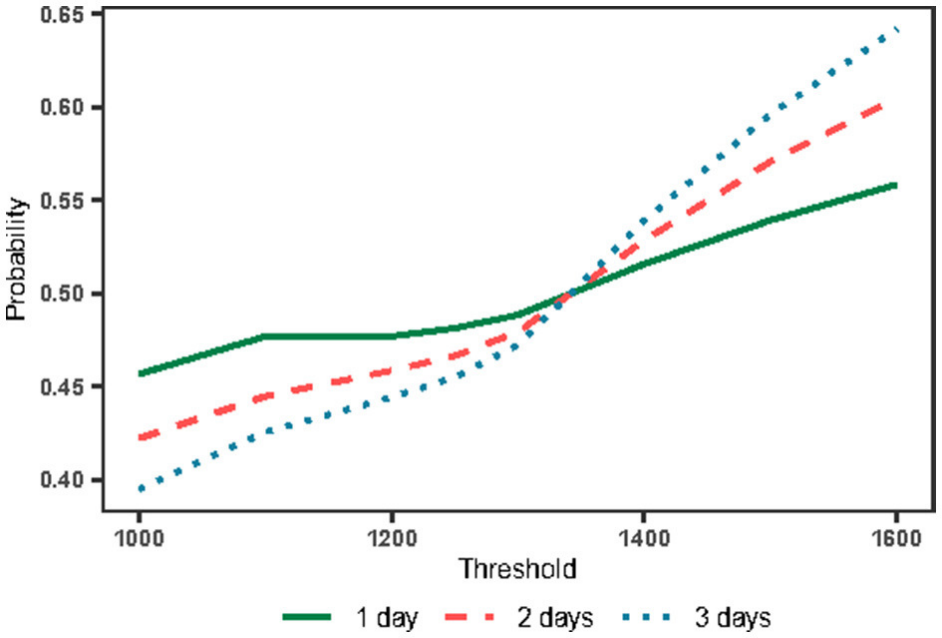
The mean probability with the change of threshold and observation days.

The second idea was to increase the capacity of the hospital, which we hoped would relieve the difficulty of healthcare accessibility for the whole population. Therefore, we changed the value of the threshold *c* to observe the trend of the mean probability *p*. Based on the previous data analysis, three conditions were selected to observe 1, 2, and 3 days, with a discount rate of 0.2. Based on the results of the processing presented in Figure 5, we can find that the trend of the entire probability*p* divided into two main phases, which are the two parts of the threshold *c* < 1,350 and >1,350.

The point where the threshold *c* = 1,350 is of great importance obviously. By analyzing the number of hospital visits in 2020, we found that 1,350 was the median number of hospital visits. In addition, we found that when choosing threshold *c* as 1,350, the mean value of *p* obtained was 0.5 regardless of the strategy chosen by the patient. This suggests that the median number of hospital visits is an important influencing parameter under this update function. However, it is difficult for patients to obtain this data.

When the threshold *c* < 1,350, the number of days that the population observes hospital visits exceeding the threshold *c* > 180 days, indicating that the population believes the overall medical resources are showing saturation. At this point, as the amount of information available to the public increases, it makes the public present a less optimistic mindset about access to care. Despite this state of affairs, the direct difference between these strategies is not significant, indicating that people maintain their confidence in going to the hospital despite the notion of the difficulty in healthcare accessibility, which is also reflected in the expected payoff equation later.

When the threshold *c* > 1,350, the public observes that the number of hospital outpatient clinics is tight for <180 days, indicating that the overall medical resources are not saturated. At this time, with an increase in the amount of information available to the public, the public is optimistic about the medical treatment situation and tends to increase optimism rapidly. The main reason for this phenomenon is that an increase in the threshold *c* increases the number of consecutive days when medical resources are abundant. Thus, it is easier to accumulate the confidence value as the number of days under observation increases.

Notably, increasing the threshold value *c* (i.e., the capacity of hospitals) can reduce the difficulty of healthcare accessibility. However, we cannot increase hospital access indefinitely for two main reasons. First, the hospital–patient game was built on the basis of analysis of the minority (patients). When the probability*p*increases, it will lead to a larger number of patients' inrush, which will probably make the number of visits exceed the threshold, thereby destroying the system's equilibrium successively. The second reason is the cost constraints of the hospital itself.

### 3.3. Expected payoff equation

We explored the balance equation in previous sections and now we need to analyze the other core equation, i.e., the expected payoff equation Eq. (14). The most central part of the expected payoff equation is the setting of the payoff *H* and *L*. Here, we use two major classes of eight items to construct an evaluation index system to comprehensively quantify the expected payoff. The measure of payoff *H* includes four indicators, including the number of beds, bed utilization rate, average number of consultations per day, and inpatient beds, while the measure of payoff *L* includes total number of consultations, number of hospital admissions, average disposable income, and average hospitalization days of discharged patients.

**Figure 6:**
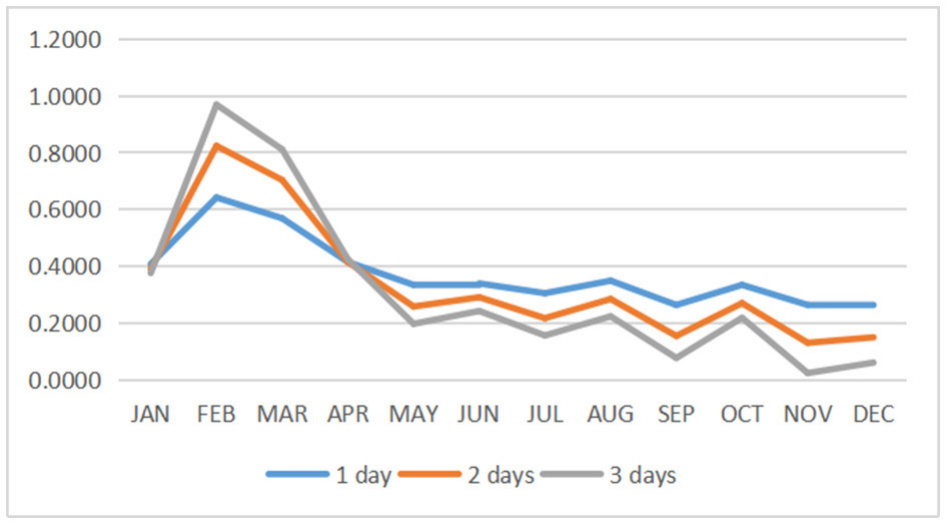
The trend of the payoff with different observation days and months.

According to the impact and importance brought by each indicator, we calculated the composite index of healthcare payoff for each indicator *via* the aggregative indicator method. The final data are presented in Table 6. By calculating the resulting payoff *H* and *L* through the aggregative indicator and combining the results of our previous calculations about the probability *p*, we can obtain the trend of the core expected payoff. Here, we select three cases of observation strategy (1 day, 2 days, and 3 days, respectively) for analysis (refer to Figure 6). From the overall trend, we can see that the expected payoff in 2020 for all three cases (i.e., from 1 to 3 days) is >0, which indicates that the hospital has brought good payoffs to the people and increased the confidence of visiting the hospital.

**Table 6:** The composite measure index for healthcare.

**Payoff category**	**Indicators**	**National data**	**Instance data**	**Evaluation standard value**	**Comprehensive index**	**Payoff**
H	Number of Beds	1,002	1,200	1.2	1.98	1.55
	Bed utilization rate	81.50%	64.63%	0.79	2.07	
	Average number of consultations per day	6.3	5.47	0.87	1.32	
	Inpatient number per day	2.1	2.9	1.38	−3.81	
L	Total number of consultations	600,801	499,438	0.83	1.69	-0.72
	Number of hospital admissions	31,285	38,775	1.24	−2.39	
	Average disposable income	32,189	30,949	0.96	−0.39	
	Average hospitalization days of discharged patients	8.6	8.28	0.96	0.37	

By looking at the data throughout 1 year, it is observed that people have higher payoffs between January and March, which is due to the fact that the hospital is not saturated during these months (i.e., the “difficulty of healthcare accessibility” phenomenon is not serious). However, people's expectations of hospital revenue tend to be close to 0 from October to December. In addition, the expectations remain positive, which indicates that people are clearly feeling the pressure to get medical treatment with satisfaction.

**Figure 7:**
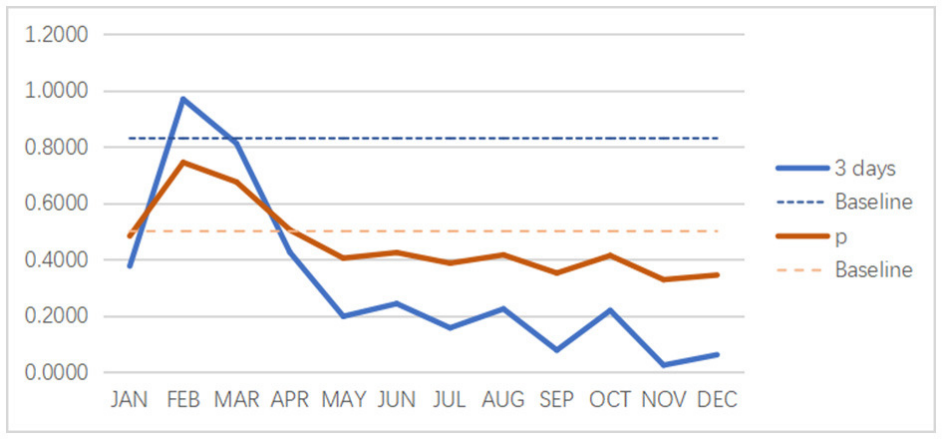
The direct relationship between the expected payoff (3 days as an example) and the balance equation.

We use *p* = 0.5 as the baseline and observe the direct relationship between the expected payoff equation and the balance equation (refer to Figure 7). We take three observation days as the analysis time period. First, we can find a positive correlation between the two equation values. Second, we find that a slight change in the *p*-value causes a significant change in the final result of the expected payoff equation, especially from August to December.

## 4. Discussion

The main purpose of this study was to investigate how the inclination of individuals seeking healthcare services is influenced by previous attendance to tertiary hospitals where the mismatch between supply and demand sides is especially prominent due to absolute or relative inadequacy of medical resources. The research on the problem of healthcare service has drawn extensive attention, models of patients, patients and doctors, and hospitals and related sectors have been widely discussed. The most grim reality in China is “difficult and expensive to seek medical services”, and patients are directly faced with medical institutions and the supplied medical services. Therefore, this study takes patient and tertiary hospital as the main object of analysis, taking into consideration of crowding and medical service level, and aims to find out the mismatch which causes patients' intuitive feeling of difficulty in seeking medical treatment.

Populations' access to healthcare is extensively addressed by the public and policymakers, with a focus on equity and efficiency. As a complex and multi-factorial construct, access to healthcare has been validated in numerous studies considering healthcare systems, socioeconomic status, and resource allocation, which have generated distinct perspectives of accessibility to healthcare. Globally, healthcare accessibility is a major concern for the public and policymakers. Healthcare accessibility mainly depends on the availability and affordability of healthcare services (26), and in turn, it influences people's psychology and behavior choice in healthcare seeking. Populations do not act through straight rational algorithms, but their decision might reflect aspects that are in contradiction with cost and benefit. As a particular action is a function of the two interacting variables, i.e., perceived benefits and perceived losses, a bad experience will increase the cost under given benefits brought by healthcare attendance in non-emergent situations.

Although health outcomes have improved in the past several decades, China, as one of the middle-income countries with a large amount of population, is faced with a contradiction between resource-constrained settings in medicine and changing health needs as well as growing public expectations, posing challenges for the public getting approach to healthcare services (4). Since the establishment of the People's Republic of China in 1949, China's healthcare system has undergone several transformations with the aim of ensuring that all sectors of society have equal and adequate access to primary healthcare, regardless of socioeconomic and geographical factors. However, existing studies suggest that most people in China believe that the system is unequal and that wealthy people and those living in cities enjoy much greater access to care than poor and rural residents in the country (12). One of the greatest challenges faced by the Chinese medical system is the highly concentrated allocation of supply-side resources at tertiary public hospitals. Compared to primary healthcare institutions, tertiary hospitals are usually well-equipped with advanced facilities and well-trained specialists (27), resulting in overwhelming workloads and long-waiting lines, which bring challenges to equality and efficiency of health resource allocation and health service utilization (28).

Different from situations in an emergency, the results show that by calculating the probability, outpatients in general situation are not overly optimistic about having good access to healthcare, which reflects the sentiment of “difficulty for healthcare accessibility”. Simultaneously, we found that when people receive more information about medical care, the perception of difficulty for healthcare accessibility will be aggravated. Second, the probability analysis reveals the basic beliefs of the public. Although they believe that the current medical efficiency is controversial and that it is difficult to access medical care, the overall efficiency of visiting the hospital is considered good. However, the overall positive benefit leads to people being more willing to visit a hospital, thereby increasing the burden on hospitals and further causing the probability to tend toward 0. The two aforementioned points jointly explain the phenomena of difficulty for healthcare accessibility from the patient's perspective.

Technological advances make it possible to get online hospital appointments for residents. Now, most of the tertiary hospitals in China use a hierarchical diagnosis and treatment system and most people arrange their attendance time at the hospital based on the arrangement through the online system. Considering the emergency degree with healthcare, there is a prioritization in life-saving treatments and medical resources allocation for emergency care, and the emergency outpatient departments are usually independent of the general outpatient departments to improve the efficiency of medical treatment. However, the overburdened workforce and equipment, as well as limited admission time caused by absolute or relative medical resources insufficiency, remain access barriers to healthcare services, further affecting patients' choice for healthcare resources utilization to a great extent. Compared to other countries, residents in China usually seek medical treatment by going to a pharmacy or hospital, not their family doctor when he or she is ill, and there appears another choice in healthcare-seeking behavior. The ranking of global medical quality shows that China has been one of the countries with the largest improvement in the quality of medical care from 1990 to 2015. The gap index in the quality of inter-regional medical services in China also narrowed (29).

Gaps in health-related investment and investment-output efficiency are also reflected in human resources for medical care, which are clearly a prerequisite for health care, with most medical interventions needing the services of doctors, nurses, or other types of health workers. The global shortfall in healthcare workers will reach 12.9 million by 2035 (30), resulting in shortages of medical personnel for hospitals, and the shortage of healthcare professionals is even more acute in China (31). The scarcity of available physicians, especially specialists, forces managers to focus on their daily scheduling and work time. Meanwhile, physician-specific aspects are usually outstanding in the field of most valuable considerations for patients (25), so another contradiction arises. As the physician scheduling is usually fixed by the day of the week for outpatient services in China, the outpatient numbers on different days of the week are extremely heterogeneous, consequently, resulting in another presentation of inequality and insufficiency.

Minimizing difficulty in healthcare accessibility helps to close the gaps for the public in healthcare seeking, and many new methods have been developed to improve such measures. Accessibility and satisfaction related to healthcare services are conceived as multidimensional concepts (4). Moreover, the selection of an appropriate model must be based on an analysis of real-world healthcare utilization behavior. When dealing with healthcare, it is important to consider the differences in each hospital or criterion. Based on the indicators adopted by other scholars and the concerns of this study, the indicators were selected from the following three aspects: outpatient services, inpatient services, and bed utilization (7, 32–34). Detailed information is included in the healthcare payoff indicator (e.g., information regarding healthcare staff qualities, quality of interaction, or helpfulness). All of these different characteristics could generate a more integral perspective of accessibility to healthcare. It is also important to mention that we generalized the concept of healthcare service supply in our study to consider the patients receiving services.

Ultimately, we used game theory to explore and solve the dilemma of difficulty for healthcare accessibility. However, we found that solving this phenomenon is not simple. Access to medical care involves a combination of a series of factors, and any change in the healthcare system has an effect on two sides. Good healthcare access and high quality of care will bring a good attendance experience and improve the actual payoff, followed by a demand increase and supply burden on hospitals, which will lower down attendance experience and tend to a new equilibrium state only if the medical resources with high quality are saturated compared to demand. The role of the regulating side appears fundamental for solving this problem, which requires stakeholders to work together, coordinate arrangements, and allocate medical resources to meet basic medical demands for residents. Based on the current situation, strengthening health education and improving the health status of residents (35), strengthening the primary care system and promoting the utilization of primary care, implementing hierarchical diagnosis and treatment, reasonably diverting the demand for medical services (3), and rationalizing the leveraged adjustment mechanism of medical insurance (36) are the main directions to solve this problem.

We chose a combined method of El Farol Bar problem and reinforcement learning to get an overview of this problem. The methodology described in this study enables us to interpret the mismatch between supply and demand sides in the face of complex interaction models and help resource allocation from the perspective of regulating side as well. Moreover, the expected payoff equation is sufficient to combine the quality of medical service into the model. In fact, the results provided particular evidence on such a question overall and this method proved to be applicable in the analysis of such situations, which can be used to conduct further research on resource management. Our illustration of this methodology in the context of difficulty for healthcare access led to several key findings. First, the population is not optimistic about going to a tertiary hospital for medical treatment overall despite the efforts devoted to promoting healthcare accessibility. Second, there is an obvious mismatch between supply and demand sides from the analysis of the data, which needs further efforts from the regulating side. Third, the expected payoff based on healthcare quality released an optimistic signal about healthcare services. Finally, the main strength of the present study lies not only in its contribution to the fundamental evidence on the contradiction between supply and demand sides of healthcare but also provides the development of mixed-method approaches with a dual-game model that incorporate concepts and techniques from different perceptions. Limitations exist in our current model, which provides directions for our future study. First, as the region where the research object is located has implemented a hierarchical diagnosis and treatment system, there is no significant difference in waiting time, so we did not take this factor into account in the model. Second, financial status and transportation problems are also prominent considerations for healthcare choice. However, the coverage of universal medical insurance reduces the financial barriers to a large extent, the migration in this city is relatively low, and the choice of healthcare facility for residents is relatively stable, which facilitate the analysis with the given model. Third, the model gives consideration to both demand and supply sides, and both players might have incomplete and/or imperfect information. In future study, we will measure the waiting time for medical treatment through questionnaires and on-site traffic statistics considering the other hospitals of different levels and apply our model in different medical specialties further.

## Data availability statement

The raw data supporting the conclusions of this article will be made available by the authors, without undue reservation.

## Author contributions

Conception or design of the study, validation, and critical revision of the article: MZ. Methodology: WW. Software: YC. Data analysis and interpretation: WW and FW. Drafting the article: FW. All authors contributed to the article and approved the submitted version.
