# Supplementary material for: Two-stage dual-game model approach to view the difficulty of healthcare accessibility
Source: Front Public Health. 2023 Mar 9;11:1078675. doi: 10.3389/fpubh.2023.1078675 (PMC10033865; doi:10.3389/fpubh.2023.1078675)
Supplement: Supplementary file 1 [file Table_1.DOCX]

Supplementary Material

# Supplementary Figures and Tables

## Supplementary Tables

#### Supplementary Table1 A description of notations

| Notation | Description |
| --- | --- |
| $N$ | The number of players |
| $w_{ij}$ | Payoff weight of player $i$ who choose action$j$ |
| $H, L$ | Payoffs that players will receive |
| $c$ | Capacity of the bar |
| $x_{ij}$ | Probability of player $\text{i}$ to choose action $\text{j}$ |
| $x_{ij}^{*}$ | Equilibrium point |
| $A_{ij}$ | The number of times that action $j$ is used by player$i$ in time interval $t$ |
| $t$ | Time interval |
| $Y_{i}$ | Probability mass function |
| $u_{ij}$ | Expected payoff for player $i$ by taking action $j$ |
|  | The state set space |
|  | The set space for actions |
|  | The set space of reward |
|  | The transition probability matrix |
| $\gamma$ | The return discount rate |
| $\tau$ | Trajectory |
| $J\left(\pi\right)$ | Expected return |
| $p(\tau\vert\pi)$ | The probability of trajectory occurrence |
| $r_{t}$ | The return of the agent at time $t$ |
| $w$ | Learning rate |
| $\text{p}_{\text{t}}$ | The probability of choosing to go to the hospital at time$t$ |
| $\text{Action}_{\text{t}}$ | The actions at time $t$ |
| $S$ | Events |

| Days of  the week | Initial value | Observation days | | | | | | |
| --- | --- | --- | --- | --- | --- | --- | --- | --- |
|  |  | 1 day | 2 days | 3 days | 4 days | 5 days | 6 days | 7 days |
| Monday | 0.5 | 0.423 | 0.362 | 0.312 | 0.273 | 0.241 | 0.216 | 0.196 |
| Tuesday | 0.5 | 0.447 | 0.405 | 0.371 | 0.344 | 0.322 | 0.305 | 0.291 |
| Wednesday | 0.5 | 0.445 | 0.402 | 0.366 | 0.338 | 0.316 | 0.298 | 0.284 |
| Thursday | 0.5 | 0.464 | 0.435 | 0.413 | 0.394 | 0.376 | 0.361 | 0.349 |
| Friday | 0.5 | 0.45 | 0.41 | 0.378 | 0.352 | 0.332 | 0.316 | 0.302 |
| Saturday | 0.5 | 0.569 | 0.621 | 0.658 | 0.684 | 0.701 | 0.711 | 0.719 |
| Sunday | 0.5 | 0.600 | 0.680 | 0.744 | 0.795 | 0.836 | 0.869 | 0.895 |

#### Supplementary Table2 The special situation with probability under different days of the week

## Supplementary Figures


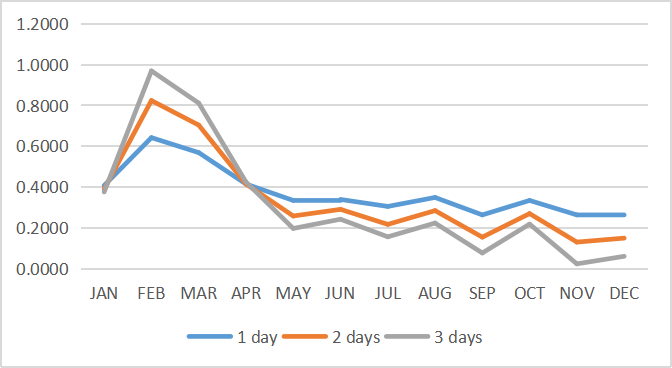


**Supplementary Figure 1.** The trend of the payoff with different observational days and months.


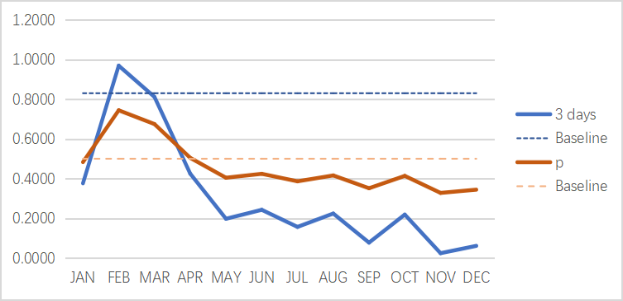


**Supplementary Figure 2.** The direct relationship between the expected payoff (3 days as an example) and the balance equation.
